# Points in Typhoid Fever

**Published:** 1883-08

**Authors:** 


### ARTICLE II.

POINTS IN TYPHOID FEVER. SERVICE OF DR. M. MANNHEIMER, ALEXIAN BROTHERS' HOSPITAL CHICAGO, DR. THOMETZ, HOUSE PHYSICIAN.

From August 1, 1882, to January 20, 1883, there were treated in the Alexian Brothers' Hospital fifty-two cases of typhoid fever. Of this number, twelve (12) were admitted in August, sixteen (16) in September, eleven (11) in October, eight (8) in November, and five (5) in December.

Most of the patients were between twenty and thirty years of age, the youngest being fourteen, and the oldest forty-five. Nearly all of them were sick from one to eight days before entering the hospital. The usual prodromic symptoms were complained of. The principal symptoms were headache, dizziness, chills, insomnia, excessive thirst, and a tired, painful feeling in limbs. The countenance usually presented a flushed appearance, and in a day or two later, a dull, stupid expression, the tongue being dry, nearly always coated, and often swollen. Epistaxis occurred in 15 out of the 52 cases; looseness of the bowels in about 40. The typhoid spots on the abdomen were found in 23 cases only. In four cases, the disease set in suddenly, with intense headache and violent delirium. For diagnosis, the symptoms usually relied upon were headache, fever, dicrotic pulse, and enlargement of the spleen, not omitting the appearance of the tongue. The spleen was found enlarged in 31 cases on admission to the hospital, and the pulse found to be dicrotic in at least the same number of cases.

Two of the patients claimed that they had typhoid fever several years ago.

During the course of the disease, the usual symptoms of typhoid fever were met with, want of appetite existing in nearly all cases during part of the disease. The characteristic pea-soup stools were found in almost every case; exceptionally, there would be no appreciable change from the stool of a healthy individual.

Tympanitis, intestinal gurgling, and iliac tenderness were also found. Slight hæmorrhage from the bowels occurred in four cases.

A subacute bronchitis existed in almost every case, hoarseness of the voice from slight laryngitis, and deafness from the dried condition of the mouth and Eustachian tube leading to the ear.

Slight delirium usually occurred in the middle of the disease, the delirium not being very violent or acute, except in those cases already mentioned, where disease commenced by delirium. Mental condition was weak, dull, and indifferent.

The pulse was accelerated, in ordinary cases, to about 90 beats per minute, in many cases not going over 90 throughout the disease. In the more severe cases, the pulse would range from 90 to 120, being at the latter figure during the height of the fever. As convalescence approached, the pulse would decline, often below normal, to 60, and in several cases to 40; the main characteristic of the pulse being its quick, vibratory and dicrotic quality.

As regards the temperature, we found a gradual rise, both in morning and evening, until a certain height was reached, where it would remain four or five days, sometimes two weeks, and then gradually decline.

There was a difference in the morning and evening temperature of from 1 to 2° F. At the height of the disease, the temperature in the evening ranged between 104° and 105°; and in the morning between 102° and 103.5.° When the fever had declined, it was not unusual to see the temperature of body rise almost suddenly to 103°, and even higher, and in a few days become normal again. A sudden rise during the course of the disease betokened some complication.

Relapses occurred in six cases, and these passed through a second career with the characteristic symptoms aggravated but of shorter duration. None of these proved fatal.

Of the complications that occurred, there were three with acute bronchitis, one with laryngitis, five with pneumonia, two with thrombosis of femoral vein, both of the left side, and one case with acute orchitis and epididymitis. Perforation of the intestine occurred in one case; the perforation on post mortem examination was found to be about eight inches above the ileo-cæcal valve.

Average number of days of treatment was twenty-six, counting from the time the patients entered until fever was gone for six to ten days, and the men able to be around the hospital.

Forty-two patients were in hospital four weeks and less, eight were from six to nine weeks, and two cases were there three months before they left.

Out of the fifty-two cases, four terminated fatally. The cause of death in one case being the occurrence of a pneumonia; one died from peritonitis following a perforation; one, aged 44, died from continued high temperature, and one death occurred in an overworked and debilitated patient.

### TREATMENT.

The object of our treatment was not to abridge the duration of the disease, but to tide the patient over it. The diet was carefully attended to; milk, eggs, and farinaceous preparations were used. About 7 A. M. the patients were allowed a cup (8 ounces) of milk with a slice or two of toast; at 9 A. M. and 3 P.M. they received a bowl of milk punch (consisting of milk, eggs, and whisky). For dinner and supper—milk, toast, and farinaceous soup. This plan of dieting suited nearly all of the patients, and rarely was it necessary to make any change.

Alcohol was used judiciously. As soon as there were any indications for its use it was administered as a supporting and stimulating measure.

Nearly all were put on a slightly acidulated mixture, ʒss of dilute muriatic or phosphoric acid in ds ℥iv of water, and ℥ss given three or four times daily. Water was freely allowed, to be taken often and in small quantities, so as not to overload and distress the stomach. Cleanliness, free ventilation and good nursing was required in the fever ward. Palliative measures were now and then called for. Protracted insomnia was relieved, and refreshing sleep procured by bromides. Excessive and exhausting diarrhœa was immediately checked by astringents in a mucilaginous mixture. Constipation relieved by injections of warm soap water.

### REDUCTION OF TEMPERATURE.

Believing that many typhoid cases would die from the continued high temperature it has been our endeavor to keep down the bodily heat. For this purpose, various methods have been tried. Quinine in 15, 20, 40 gr. doses have been tried and with good results in a few cases. In two patients, whose evening temperature, was 105° and 106°, 30 grs. of quinine in one dose reduced the temperature to 98° F., and no subsequent rise. But most of the patients objected to the quinine, vomited it up, or complained of headache upon its use. Digitalis and quinine together were sometimes used. Sponging the body was resorted to in some cases.

Packing the body in the wet sheet and sprinkling with cold water reduced the temperature from one-half to two degrees in from three to four hours; the temperature would rapidly rise on removing patients from the sheets.

Salicylate of soda was used, and by far the best results were obtained from its use. The drug was exhibited in doses of 15 grains every ½, 1, 2 or 3 hours, depending on the temperature. If the temperature was 103° or higher in the morning, two doses were given ½ hour apart, the third dose an hour later, and if a fourth dose was necessary, it was given 1 or 2 hours later, until the effect of immediate reduction in temperature followed. After the first, or at least second dose, the dry, burning skin becomes moist, the rapidity of the pulse lessens. After using it two or three days, the dry, parched tongue becomes wet, the black fur gradually disappears, and the patient gets a ravenous appetite.

In three quarters of our cases, we have used the salicylate to keep down the temperature, and find it to be better than quinine or any form of wet packing. It is easily taken, does not disturb the stomach as often as is thought, and does not weaken the patient. Only one patient out of about forty who used it, complained of headache from its use.

When several doses were used in quick succession, a tablespoon of whisky was given with each. To show the action of the remedy, a copy of one of the records is subjoined:

A. D., age 25, entered hospital in evening, with temperature of 103.4°. In morning at 9 o'clock, temperature was 101.4°; at 10, 11 and 12 he took 15 grains of sodic salicylate; at 2 P. M. temperature was 101.2°, and 6 P. M., 100.4°. Next morning, temperature was 101.4°; at 2 P. M., 103.2°; and at 2:30, 3:30 and 5, 15 grains were given; at 6 P. M., temperature was 101.2°. Next day no salicylate was used, and the evening temperature was 103.5°. These figures speak for themselves.

If one drachm of the drug is used in one dose, its effects are not as good and as persistent as when given in 4 divided doses.

It seems that by its use, the duration of the disease is not as long as without its use; the patients seemed improved in every way; they felt better and looked better. They convalesce rapidly. Why it acts so favorably, we cannot say, unless it should be that some form of poison in the blood is rapidly eliminated through the skin and kidneys. We could find nothing better than it, and nothing equal to it, unless it should be the judicious and frequent use of cold baths, and these cannot be given properly where there are many patients.

